# In Vitro Wound Dressing Stack Model as a First Step to Evaluate the Behavior of Dressing Materials in Wound Bed—An Assessment of Mass Transport Phenomena in Hydrogel Wound Dressings

**DOI:** 10.3390/ma14247702

**Published:** 2021-12-13

**Authors:** Ewelina Baran, Anna Górska, Artur Birczyński, Wiktor Hudy, Wojciech Kulinowski, Witold Jamróz, Władysław P. Węglarz, Piotr Kulinowski

**Affiliations:** 1 Institute of Technology, The Pedagogical University of Kraków, Podchorążych 2, 30-084 Kraków, Poland; ewelina.baran@up.krakow.pl (E.B.); artur.birczynski@up.krakow.pl (A.B.); wiktor.hudy@up.krakow.pl (W.H.); wojciech.kulinowski@up.krakow.pl (W.K.); 2 Department of Pharmaceutical Technology and Biopharmaceutics, Faculty of Pharmacy, Jagiellonian University Medical College, 9 Medyczna Street, 30-688 Kraków, Poland; witold.jamroz@uj.edu.pl; 3 Department of Magnetic Resonance Imaging, Institute of Nuclear Physics, Polish Academy of Sciences, Radzikowskiego 152, 31-342 Kraków, Poland; wladyslaw.weglarz@ifj.edu.pl

**Keywords:** cryogels, hydrogels, mass transport, diffusion, moving fronts, magnetic resonance relaxometry, magnetic resonance imaging, wound dressings, in vitro model, interfacial phenomena

## Abstract

Wound dressings when applied are in contact with wound exudates in vivo or with acceptor fluid when testing drug release from wound dressing in vitro. Therefore, the assessment of bidirectional mass transport phenomena in dressing after application on the substrate is important but has never been addressed in this context. For this reason, an in vitro wound dressing stack model was developed and implemented in the 3D printed holder. The stack was imaged using magnetic resonance imaging, i.e., relaxometric imaging was performed by means of *T_2_* relaxation time and signal amplitude 1D profiles across the wound stack. As a substrate, fetal bovine serum or propylene glycol were used to simulate in vivo or in vitro cases. Multi-exponential analysis of the spatially resolved magnetic resonance signal enabled to distinguish components originating from water and propylene glycol in various environments. The spatiotemporal evolution of these components was assessed. The components were related to mass transport (water, propylene glycol) in the dressing/substrate system and subsequent changes of physicochemical properties of the dressing and adjacent substrate. Sharp changes in spatial profiles were detected and identified as moving fronts. It can be concluded that: (1) An attempt to assess mass transport phenomena was carried out revealing the spatial structure of the wound dressing in terms of moving fronts and corresponding layers; (2) Moving fronts, layers and their temporal evolution originated from bidirectional mass transport between wound dressing and substrate. The setup can be further applied to dressings containing drugs.

## 1. Introduction

The term ‘wound’ can be defined as any type of damage or disruption to the normal anatomical structure and function that can range from a simple break in the continuity of the epithelial lining of the skin to other deeper structures such as tendons, muscles, vessels and even bone [1]. Regardless of the type of wounds, the primary goal in their management is to achieve healing as quickly as possible with optimal functional and aesthetic results. A part of this process includes wound dressing, designed mainly to be in contact with the wound and provide a physical barrier to protect from further trauma or contamination [2,3]. Bearing in mind that no single dressing is suitable for all stages of healing and all types of wounds, there is still a need for the development of materials that accelerate wound healing in specific clinical applications. The choice of appropriate dressing is crucial for effective wound management and depends on the wound’s characteristics, mainly an abundance of exudate and the stage of the healing process (hemostasis, inflammation, cell migration/proliferation, and maturation) [3]. Furthermore, a selection of an appropriate dressing requires some knowledge about the specific properties of products, taking into account, for example, adhesion, conformability and fluid handling properties. In turn, a good knowledge of the product generally depends on the quality of available research methods that evaluate the properties and efficacy of such wound care products.

In vitro tests are used regularly and aim to evaluate the functionality of prototypes of novel wound dressings, especially at the initial stage of their development. The popularity of in vitro tests is primarily due to their simplicity, low-cost, and easy standardization, as well as the absence of stringent ethical scrutiny and considerations compared to working with laboratory animals or humans. However, a limitation of in vitro testing is that the quality of studies evaluating the efficacy of wound dressings is frequently not relevant when related to the clinical situations [4].

The main European standards for the laboratory-based testing of wound care products are a series of EN 13726 standards in compliance with the EU Directive 93/42/EEC, which regulates laboratory test methods for the ability to adapt to the shape of the body, adherence, fluid handling, absorbency, breathability and other physical characteristics of wound dressings [5,6]. These laboratory-based tests ensure a fast classification of the new materials as promising or not for further clinical research. Moreover, they are used as tools to help differentiate one product from another [4]. However, it should be emphasized that not all of the parameters specified in the EN 13726 standards are appropriate for the determination of each type of dressing, and the scope of the tests described does not fully exhaust the issues related to the recognition of their usable and functional properties. To extend the scope of the standard tests, in vitro models have been developed to study the mechanisms involved in microbial biofilm formation, as bacteria in the chronic wound exist mainly in the form of biofilms [7,8,9,10,11]. For example, Werthén et al. developed an in vitro model in which biofilms can develop in the absence of a solid surface but in the presence of simulated wound fluid (containing 50% fetal calf serum and 0.1% peptone) and a collagen matrix [10]. In turn, Greenman et al. constructed a model of a moderately exuding wound to evaluate the antimicrobial activity of dressings [11]. This in vitro model consisted of cellulose discs dosed with test microbes suspended in 50% fetal calf serum. Most of these models have the advantage of being simple, but none of them correspond to the research concept focused on the cognition of interfacial phenomena at the dressing/substrate contact plane. Both standard laboratory tests and existing in vitro models do not make it possible to understand the interfacial phenomena in the dressing during its contact with the wound surface (e.g., with exudate). Therefore, there is a constant need for the development of simple in vitro models, which could mimic, to some extent, the wound environment and enable a study of changes in physicochemical properties across the dressing when applied to the wound.

Magnetic resonance imaging (MRI) can be used for soft tissue imaging, e.g., for wound diagnostics and monitoring [12]. In the context of wound dressings, MRI was mainly used for testing the safety and MRI compatibility (e.g., distortions) of silver-based dressings [13,14]. In vitro testing of wound dressings using an MRI technique as such has been marginal so far, but published studies revealed the potential of these techniques. Previous papers by Górska et al. have consistently emphasized the usefulness of MRI for the assessment of important features of hydrogel wound dressings [15,16]. On the example of asymmetric wound dressings obtained by the freezing-drying method, a quantitative assessment of various, spatiotemporal aspects of wound dressing membrane hydration has been presented. It has been possible to assess the time required to hydrate the membrane to be applicable as a wound dressing (time to reach steady-state) and the period in which the hydrated wound dressing retains its desired “working” parameters (i.e., structural and molecular properties) [15]. Magnetic resonance imaging has also been used as one of the methods of characterization of hydrogel wound dressings obtained by the freezing–thawing (F-T) technique. It has been possible to catch subtle changes inside the membrane during contact with bulk water, e.g., assessment of mass transport-related phenomena at the molecular- (detection of propylene glycol, confinement effects related to pore size) as well as at the macro-level (swelling) [16]. Nevertheless, these in vitro studies did not seem to have much in common with dissolution testing or with real application conditions.

Taking into account the unmet need for the development of new research methods, which could mimic to some extent the wound environment and previously assessed the potential of nuclear magnetic resonance, especially MRI methods, the main goal of the study was:

- to develop a simplified in vitro model simulating the place of application of a dressing to a wound area;
- to assess the potential of magnetic resonance imaging for studying changes in dressing properties when applied on various substrate environments.

The model should enable the assessment of developed materials in somewhat more realistic conditions. In the current manuscript, a method was proposed that allows an understanding of the phenomena accompanying the application of a dressing in the wound bed area, observed in situ without disturbing the processes taking place inside this material. The emphasis was put on interfacial phenomena at the dressing/substrate contact plane. To show the usefulness of the method, the assessment was made using samples of two previously described PVA-based hydrogels with and without the addition of Labrasol^®^ used as a surfactant and solubilizer in topical formulations [16]. Moreover, two different substrate environments were used, i.e., fetal bovine serum (medium related to in vivo situation) and propylene glycol as one of the possible acceptor fluids used for in vitro drug release tests [16]. According to our knowledge, the use of MRI to characterize wound dressings in contact with different environments and the analysis of the mechanisms occurring at the dressing–wound interface has not been reported as yet.

## 2. Materials and Methods

In the present work, the following materials were used: polyvinyl alcohol (PVA, Mowiol^®^ 56–98, Mw ~195.000 Da; degree of hydrolysis 98.4 ± 0.4 mol%) obtained from Merck KGaA (Darmstadt, Germany); propylene glycol (PG) (Chempur, Piekary Śląskie, Poland); Labrasol^®^ (L) kindly donated by Gattefossé (Saint-Priest, France); gamma-irradiated fetal bovine serum (FBS) (Thermo Fisher, Waltham, MA, USA); sodium chloride (Avantor Performance Materials, Gliwice, Poland); calcium chloride dihydrate (Chempur, Piekary Śląskie, Poland).

### 2.1. Hydrogel Wound Dressing—Preparation

The PVA-based hydrogel wound dressings were prepared by a repeated freeze-thawing cycle [16]. In brief, the samples denoted in this paper as M8 were prepared by allowing polyvinyl alcohol (8% *w*/*w*) to dissolve completely in distilled water at 90 °C under continuous stirring. The obtained solution was cooled and water and PG were added at concentrations of 82% and 10% (*w*/*w*), respectively. In order to remove air bubbles, the mixture was kept at room temperature (RT) overnight. Afterwards, the aqueous solution of PVA was placed in a Petri dish of 28.5 mm in diameter and exposed to 6 freeze-thawing cycles using an ultralow temperature freezer (Nuaire NU-9483E, Plymouth, MN, USA). In the first cycle, the samples were frozen and kept at −80 °C for 24 h; next, they were thawed at RT for 1 h. In the remaining cycles, the freezing and thawing time was 1 h (−80 °C/~22 °C).

Hydrogels with Labrasol^®^ (denoted as M8_L) were formulated according to the same procedure except that, after PVA solution preparation, the Labrasol^®^ was also added to the solution. In this case, the composition was as follows: PVA—7.8%, L—2.1%, PG—9.8%, water—80.3% *w*/*w*.

### 2.2. Wound Dressing Stack Model

**Figure 1:**
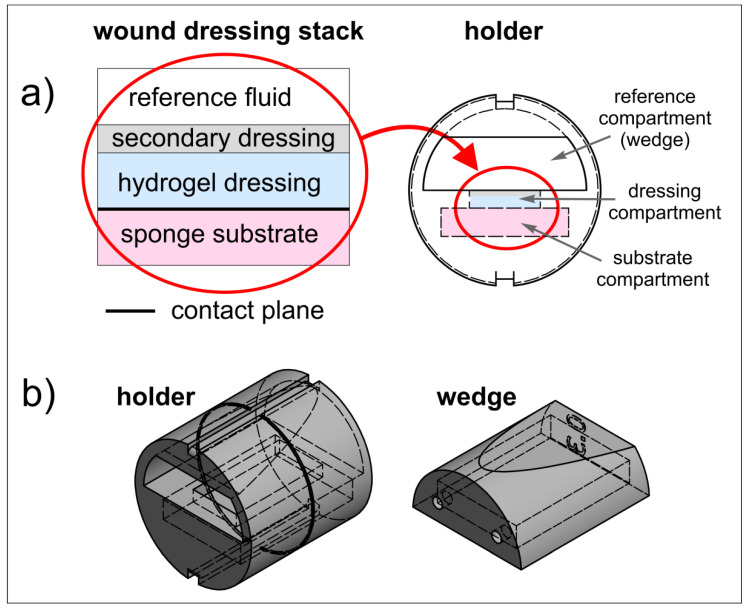
(**a**) Idea of wound dressing stack model and holder; (**b**) model of the setup (holder with a wedge) for 3D printing.

The proposed wound dressing stack model had a sandwich structure and mimicked a typical layered structure which could be found when the hydrogel wound dressing is applied to the wound area (Figure 1a).

The lowest layer was a melamine foam sponge substrate moistened with two types of media: diluted fetal bovine serum—1.5 mL, as a substitute for human wound fluid, or PG—1.5 mL for comparison purposes as a potential dissolution medium (see Górska et al., 2021 [16]).

FBS was diluted to reduce the viscosity by mixing this medium with a solution prepared from 142 mM NaCl and 2.5 mM CaCl_2_·2H_2_O in distilled water (1 L volumetric flask) with a weight ratio of 1:1. Such a dilution has already been used by Milne et al. to investigate the influence of different dressings on the pH of the wound environment [17]. For the purposes of this paper, the general term fetal bovine serum (FBS) will be used for diluted fetal bovine serum.

The second layer of the model was 10 × 20 mm^2^ PVA-based hydrogel dressing samples. Since hydrogel wound dressings are non-adhesive, are often difficult to secure, and can easily dehydrate, they required secondary dressings, usually moistened bandages. Therefore, the next layer was gauze swabs soaked with saline solution (0.9% NaCl).

For the purpose of this paper, the general term: “in vitro wound dressing stack model” will be used when describing the model simulating the place of application of a dressing to a wound area.

### 2.3. 3D Printed Holder for Wound Dressing Testing

A dedicated holder was designed to properly fix the wound dressing stack and to enable its positioning inside the MRI scanner. Holder design is presented in Figure 1a (right) and Figure 1b. It consisted of the cylindrical holder body and the wedge. In the lower part of the horizontally placed cylinder, a compartment for sponge substrate was designed (substrate compartment). This compartment had a rectangular window of the dressing sample size, which enabled wound dressing placement directly on the sponge substrate. The window was denoted as dressing compartment. The wedge was designed to assure contact between wound dressing and sponge substrate, as well as sealing the lower compartment by slight pressing on the wound dressing. The wedge was an empty structure with small holes which enabled filling with reference medium (reference compartment). The reference medium (water) served for magnetic resonance (MR) image calibration. After placement of the wound dressing sample and application of the secondary dressing, the wedge was inserted into the holder body and the holder was ready for MR imaging.

The holder was designed to be manufactured using the 3D printing technique. The holder design was made using the Autodesk Inventor Professional 2019 software (Autodesk Inc., San Rafael, CA, USA). The dimensions of the holder were matched to fit the 30 mm ID RF coil of the MRI scanner. The holder was printed using UV-Curing resin Jamg He 405 nm 3D Classical Resin super low odor (green) (Shenzhen, China) with a liquid-crystal display (LCD)-based SLA printer Anycubic Photon S (Shenzhen, China) working at a wavelength of 405 nm. UV exposition of the first three slices was 45 s and for the subsequent slices it was 30 s.

### 2.4. Magnetic Resonance Imaging and Image Analysis

MR imaging study was carried out at room temperature using 9.4 T Bruker Biospec MRI scanner (Bruker, Ettlingen, Germany) and multi-slice multi-echo (MSME) pulse sequence. The parameters of the MSME sequence were as follows: echo time (TE) = 3.644 ms, number of echoes (NE) = 256, repetition time (TR) = 5 s, acquisition buffer length (SI) = 256, phase steps = 192, number of accumulations (NA) = 1, slice thickness = 1.5 mm, field of view (FOV) = 24 × 24 mm^2^. After reconstruction, a series of 256 × 256 images at each echo time (n*TE) were obtained, with an in-plane resolution of 0.094 mm.

MR imaging measurements were performed every 15 min during the first 2 h after the sample was applied to the moistened sponge (for both sponge substrate media—FBS as well as PG). For the model with PG, additional measurements were also made after 24 h and 48 h. In this case, processes at the wound dressing/substrate contact plane were much slower. When the FBS was used, a noticeable lack of further changes was observed after 2 h. It should be noted that FBS has limited stability and changes its properties approximately 10 h after thawing.

The MSME image stacks (images obtained at consecutive echo times) were imported to Fiji distribution of ImageJ version 1.44 (National Institutes of Health, Bethesda, ME, USA, http://rsb.info.nih.gov/ij/, last accessed on 26 October 2021). The 10 pixel-wide (1.1 mm) segment of the image across the dressing was chosen. The rows of the resulting images were averaged to obtain profiles across the membrane and sponge substrate, i.e., 1D images perpendicular to the membrane surface. The pixel-by-pixel analysis of the 1D image stacks was performed to obtain 1D parametric images of effective *T*_2_ decay constant and signal amplitude *A*. For each profile, pixel image intensity vs. echo time, up to three signal components were fitted using the Levenberg–Marquardt algorithm with mono-, bi- or tri- exponential function, as in Equation (1).

(1) $$f\left(t\right)=y_{0}+\sum_{i=1}^{n}A_{i}e^{-\frac{t}{T_{2}{}_{i}}},$$

where *y*_0_ is the constant level, *A_i_*—amplitude of *i*th component, *T*_2*i*_—spin-spin relaxation time of an *i*th component, *n* = 1, 2 or 3. Quality of measurement setup (i.e., pulse sequence parameters adjustment, probe matching and tuning) was checked using water signal from reference compartment of the holder (amplitude of this signal in consecutive scans remained constant).The calculations were made using OriginPro 2021b (OriginLab Corporation, Northampton, MA, USA).

Regularized inverse Laplace transform to produce the distribution of *ln*(*T*_2_) was also performed for signals obtained from some specific spatial locations. Data analysis was performed using the application Inverse Laplace Transform in NMR implemented in OriginPro 2021b software (OriginLab Corporation, Northampton, MA, USA).

## 3. Results and Discussion

### 3.1. Qualitative Overview

For the purpose of the study, two previously developed hydrogel matrices were used [16]. Analyzed dressings were in the form of elastic sheets and were based on fully hydrolyzed polyvinyl alcohol (PVA), PG and water (formulation denoted as M8). The second formulation, denoted as M8_L, had the same composition but with the addition of Labrasol^®^. PVA was used to produce a spongy matrix due to its possibility of being crosslinked through an easily-conducted freeze–thaw method without the use of a crosslinking agent that could be toxic [18,19]. The usage of the PVA-based hydrogels in wound dressing applications is particularly advantageous [20,21]. When applied as wound dressings, the PVA hydrogels maintain the moist environment and absorb the exudates from the wound surface, which promotes autolytic debridement and gives a pleasant cooling and analgesic effect [22]. Furthermore, due to the non-adhesive properties of the PVA hydrogel dressings, they could be easily changed without causing new wound injuries [23]. In turn, PG is a hygroscopic substance that resists evaporation and rapid dehydration of hydrogel wound dressing. Labrasol^®^ is a surfactant and solubilizer used in topical formulations. Additionally, because Labrasol^®^ is a surfactant, it can potentially be used to facilitate wound cleansing and aid autolytic debridement.

**Figure 2:**
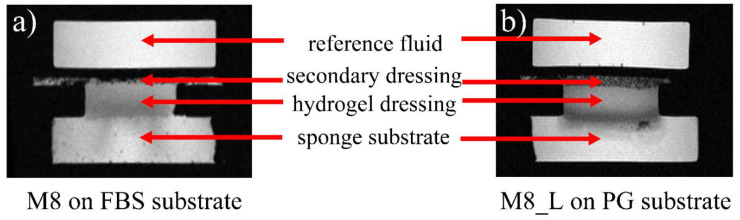
Images obtained at 20th echo (70 ms) at 15 min with MRI MSME sequence for M8 dressing samples on FBS wetted substrate (**a**) and M8_L dressing on PG wetted substrate (**b**).

Figure 2 shows an example of MR images obtained during the experiment using the proposed in vitro wound dressing stack model with a spatial resolution of about 0.1 mm. The MSME sequence enabled signal acquisition, starting from an echo time of 3.5 ms. Only volumes of the sample containing mobile protons could be imaged, i.e., water in the wedge (used as reference), water and PG inside the wound dressing, as well as water or PG in the sponge substrate. The black areas (noise level) represent parts of the 3D printed setup as well as voids (i.e., air, also air forming bubbles present in a sponge or gauze). The images show that even using qualitative results (non-parametric, *T*_2_ weighted images as in Figure 2) it was possible to observe processes occurring in the vicinity of dressing/sponge substrate contact. These processes were manifested with regions of different image intensities. Moreover, it was apparent that these processes differ when using different media for sponge substrate moisturizing (FBS or PG).

### 3.2. Quantitative Analysis

**Figure 3:**
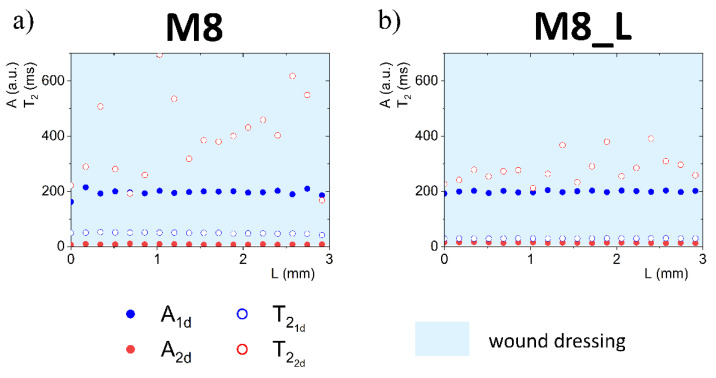
1D parametric profiles in terms of *T*_2_ relaxation time and signal amplitude (*A*) across the wound dressing before application on sponge substrate, for M8 (**a**) and M8_L (**b**) samples.

**Figure 4:**
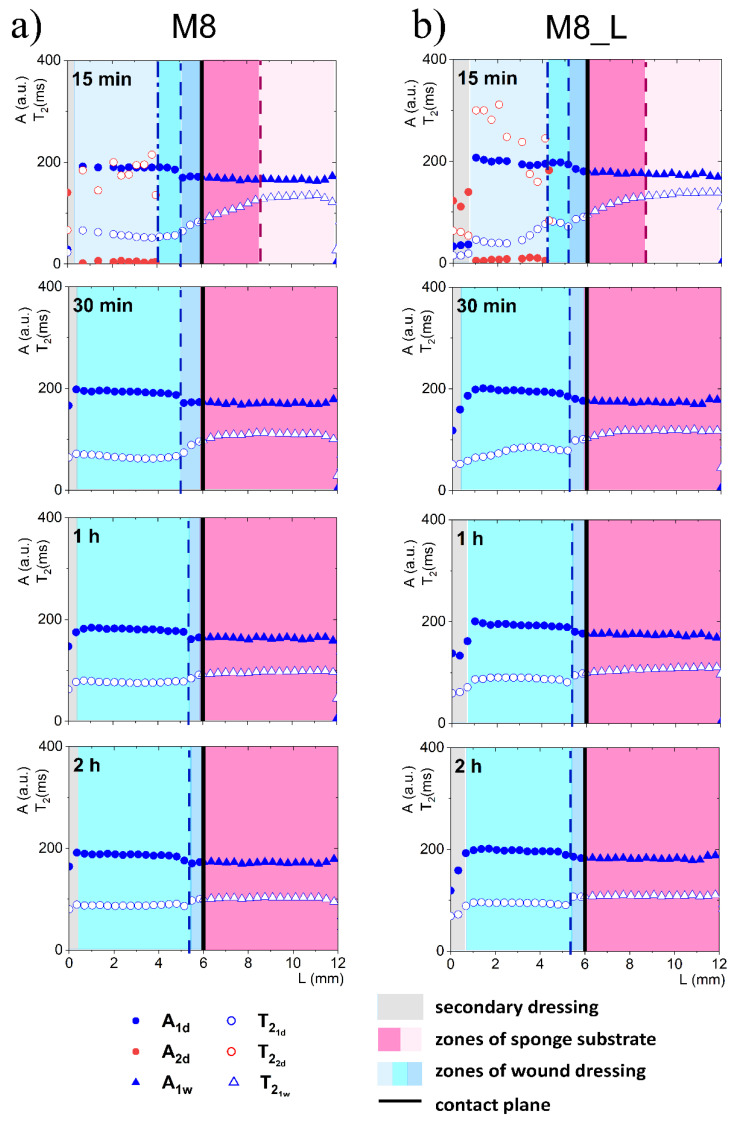
1D parametric profiles in terms of relaxation time (*T*_2_) and signal amplitude (*A*) across the simulated wound dressing stack after application on sponge substrate wetted with FBS, for M8 (**a**) and M8_L (**b**).

Complete quantitative results and interpretation of multi-echo imaging are presented in Figures 3, 4, 6 and 7. The figures present 1D parametric images (profiles) in terms of calculated *T*_2_ relaxation times and relating amplitudes (*A*) of particular signal components. The *L* denotes the distance from the top of the dressing stack (*L* = 0 mm). *L* increases down the dressing towards and across the sponge substrate. Position of dressing/sponge substrate contact plane in Figures 4, 6 and 7 (*L* = 6 mm) is marked with a thick continuous line. To check the difference between the tested materials, profiles of proton fractions across the hydrogel wound dressings (as detected in terms of *T*_2_ and corresponding amplitudes *A*) were measured before application of the dressings to the FBS or PG moistened sponge substrate. Profiles across the M8 and M8_L dressings are presented in Figure 3. Average values of the parameters as measured across the dressing are presented in Table 1. Two sources of proton pools were present in dressings. The amplitude ratio roughly reflected the water to PG concentration ratio. A pool of higher signal intensities (amplitude *A*_1*d*_) suggested that the origin of these protons was loosely bound water in the sample. Signals of a lower intensity originated from PG protons (*A*_2*d*_). In the case of sample M8, the mean amplitude for water protons (*A*_1*d*_) had a value of 195 a.u. and was close to the value obtained for sample M8_L (*A*_1*d*_ ≈ 199 a.u.). $T_{2_{1d}}$ relaxation times for this pool of protons differed and were about 49 ms for M8 and 30 ms for M8_L. This suggested a similar amount of water in both samples, but of different mobility. Average signal amplitudes and *T*_2_ relaxation times of PG protons were *A_2d_* ≈ 8 a.u, $T_{2_{2d}}$ ≈ 394 ms for M8 and *A_2d_* ≈ 14 a.u., $T_{2_{2d}}$ ≈ 297 ms for M8_L. The *T*_2_’s of both components in the M8_L were shorter (30 vs. 49 ms and 297 vs. 394 ms for M8_L and M8 respectively). This suggested that dressings contained water and glycol molecules that were tied more tightly and/or that diffusion was more restricted by the PVA network. Moreover, this was consistent with previously obtained results, which show differences between the formulations of designed hydrogel wound dressings (e.g., smaller pores in M8_L formulation) [16]. The research has shown that the addition of Labrasol^®^ enhances the mechanical strength of the dressing, which can be beneficial, as a low tensile strength is one of the most important drawbacks of hydrogel wound dressings [24]. It should also be noted that the spatial distribution of water protons was relatively homogeneous in both cases (*T*_2_ relaxation times and amplitudes were similar across the entire cross-section of the dressing), while PG protons were not. The M8 dressing has a larger spatial relaxation time dispersion than M8_L—the standard deviation of the $T_{2_{2d}}$ in M8 was 157 ms, and for M8_L was 59 ms (χ^2^ of fitting > 0.98). It is likely that Labrasol^®^ caused more spatially uniform interactions of water and PG and greater integration within the network (this was indicated by the shorter *T*_2_ relaxation times in M8_L), which is consistent with conclusions of work by Górska et al. (2021) [16]. 

### 3.3. Dressings on FBS Wetted Substrate

When considering a model with FBS wetted sponge substrate (Figure 4), fast spatial changes in the wound dressing properties took place from the very beginning of its application on the sponge substrate. These changes were related to two-way diffusional mass transport. Both the release of liquid to the sponge substrate, as well as absorption of liquid from the sponge substrate, occurred. Starting 15 min after application on the sponge substrate a narrow transient zone in the wound dressing was established with increasing *T*_2_ of shorter (water) component towards the contact with the sponge substrate. The beginning of this zone was at *L* = 5.7 mm and this front is marked with a dashed line in Figure 4. Another characteristic spatial feature was a kind of moving front, i.e., a spatial position where the long component characterized by $T_{2_{2d}}$ (PG) became undetectable. It is marked with a dash-dotted line in Figure 4. This feature suggested that PG diffused to the PG-free substrate creating a PG-depleted zone inside the dressing. Simultaneously, a slight increase in *A*_1*d*_ amplitude and $T_{2_{1d}}$ of the water component was observed, suggesting diffusion of the water (the main component of FBS) into the dressing.

At about 30 min after applying the hydrogel wound dressing to the sponge substrate, the long component inside the wound dressing completely disappeared and the process leading to flattening profiles across both dressing and the substrate finished at about 1 h. Starting from about 1 h, the changes inside the wound dressing were slow and occurred evenly across the dressing. Both in M8 and M8_L samples, an increase in *T*_2_ and amplitude were observed. This phenomenon was related to further diffusion of the FBS protons into the dressing and depletion in PG. The PG component was then undetectable, but changes in the matrix were reflected in the shorter (water) component. In comparison, in the M8 sample, an increase in $T_{2_{1d}}$ was observed at approximately half of the dressing thickness (*L* about 2 mm) from about 53 ms after 15 min to 75 ms after 1 h and to 87 ms after 2 h (i.e., by about 65% compared to 15 min). In the M8_L sample, an increase in $T_{2_{1d}}$ was noticed from about 38 ms to 90 ms after 1 h and to 95 ms after 2 h (i.e., by more than 100% compared to 15 min).

On the other hand, changes in the FBS-soaked sponge substrate were also detected after application of the hydrogel wound dressing as a result of mass transport phenomena. In the substrate region (below the contact plane between the dressing and sponge substrate), a media exchange was observed. It corresponds to the general requirements for ideal dressing materials. It especially fits the desired properties of hydrogel wound dressings, of which the most important effect after application is the creation of a beneficial skin microclimate by maintaining a proper moisture balance in the wound bed [25]. In the first 15 min, the $T_{2_{1w}}$ time of the FBS in the sponge substrate decreased towards the contact. At 15 min this zone of spatial decrease in $T_{2_{1w}}$ extended from contact plane to *L* = 5.1 mm for the M8 sample (marked by a dashed line in Figure 4) and to *L* = 5.3 mm for the M8_L sample. The shortening of the relaxation time resulted from the diffusion of PG into the substrate. It could be also hypothesized that $T_{2_{1w}}$ shortening could be partially the effect of diffusion of dissolved PVA. The reduction of the $T_{2_{1w}}$ of the substrate FBS solution in time for the M8 sample was about 23% (133 ms after 15 min to 103 ms after 2 h); for the M8_L sample it was about 19% (134 ms after 15 min to 109 ms after 2 h). On the other hand, the signal amplitude in the moistened sponge slightly increased and the signal intensity increased by about 4% within 2 h for both samples. Thus, a two-sided exchange of media at the interface between the substrate and the dressing was observed and in consequence mass transport-induced changes in spatiotemporal properties of the dressing were assessed. Such spatially resolved studies have not been performed previously for dressing materials but spatial changes in dressing properties could not be surprising. Millon et al. have shown that the porous structure of the PVA-based hydrogels, consisting of amorphous regions and crystalline regions, allowed the diffusion of molecules from this matrix [26]. Hickey and Peppas have shown that the diffusion of solutes from the PVA hydrogels prepared by freezing-thawing cyclic processing (F-T) is related to the mesh size [27]. In turn, Wan et al., in their studies on such materials, showed that the release of bovine serum albumin was mainly by diffusion [28]. Considering the analyzed materials in terms of their usefulness as a potential wound dressing, the phenomenon of media exchange seems to be beneficial. It is generally thought that the ideal wound dressing should be able to absorb excess exudate and at the same time release moisture to the wound area and thus create an optimal moist environment [29].

### 3.4. Dressings on PG-Wetted Substrate

The next part of the study concerned the same samples (M8 and M8_L) using the developed wound dressing stack model but with sponge substrate wetted with PG. As wetting with FBS made the model closer to the in vivo situation, moisturizing with PG (potential dissolution medium [16]) reflected situations that were possible when testing drug release from the dressing in diffusion cell apparatus. The knowledge of mass transport-related phenomena in such configurations would be also of great importance, e.g., it could support the understanding of the release of an active substance.

**Figure 5:**
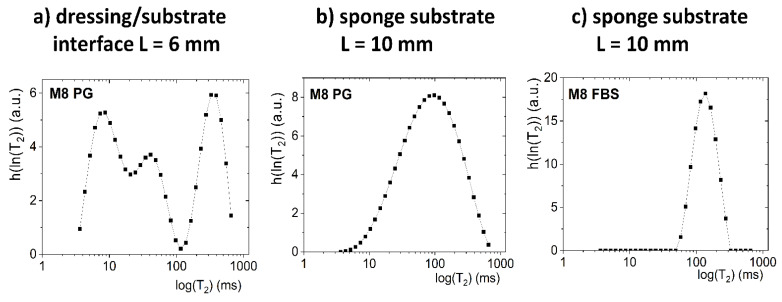
The Laplace inversion of the data obtained at 15 min after application on the substrate for: (**a**) M8 sample on PG and ROI placed at the dressing/substrate interface (*L* = 6 mm); (**b**) M8 sample on PG and ROI in sponge substrates concentrated at around *L* = 10 mm; (**c**) M8 sample on FBS and ROI in a sponge substrate concentrated at around *L* = 10 mm.

Description of the mass transport-induced phenomena occurring after application of a dressing to a sponge substrate moistened with PG was more complicated than in the case of an FBS solution. The contact between dressing and sponge substrate for M8 and M8_L samples, as previously stated, was about *L* = 6 mm. Unlike in FBS-wetted sponge substrates, two exponential components were distinguished in the signal originating from the sponge substrate moistened with PG (e.g., they were clearly visible above *L* > 10 mm for 15 min). However, these components were related to interactions between PG molecules and interactions of its molecules with the porous structure of the sponge substrate. To prove the claim, a pure PG volume sample (without a sponge) was also measured. For the PG volume sample, mono-exponential decay of the MR signal was obtained. Next, the Laplace inversion of the signal taken from ROI localized in PG-wetted substrate at around *L* = 10 mm was performed (Figure 5b). The obtained distribution was single modal, but with a long tail towards long *T*_2_ values on a linear scale (broad range of *T*_2’_s up to 600 ms) and broad *T*_2_ distribution on a logarithmic scale. The signal could be reasonably fitted by a discrete bi-exponential function (Equation (1))—one component with a short *T*_2_ relaxation time (*T*_2_ ca. 100 ms) of high amplitude, and a second with long *T*_2_ (*T*_2_ ca. 400 ms) and a low amplitude. Therefore, the initial presence of two discrete PG components in the sponge was assumed in further analysis. One can compare the result with an FBS-wetted substrate. In the case of FBS *T*_2_, the distribution obtained by Laplace inversion was narrow (Figure 5c) and extended over less than one decade, while for PG it extended over more than two decades.

**Figure 6:**
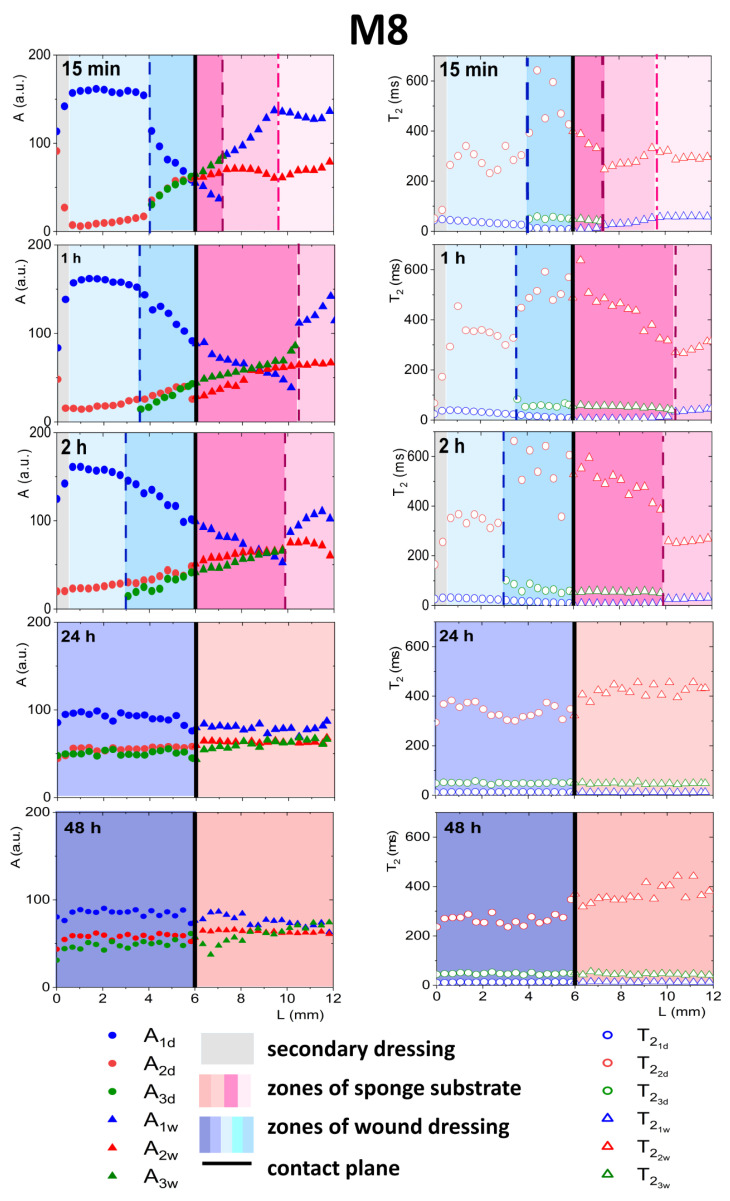
Parameter profiles obtained by MRI, relaxation time (*T*_2_) or signal amplitude (*A*), for M8 for the whole in vitro wound dressing stack model, when the sponge medium solution was PG.

**Figure 7:**
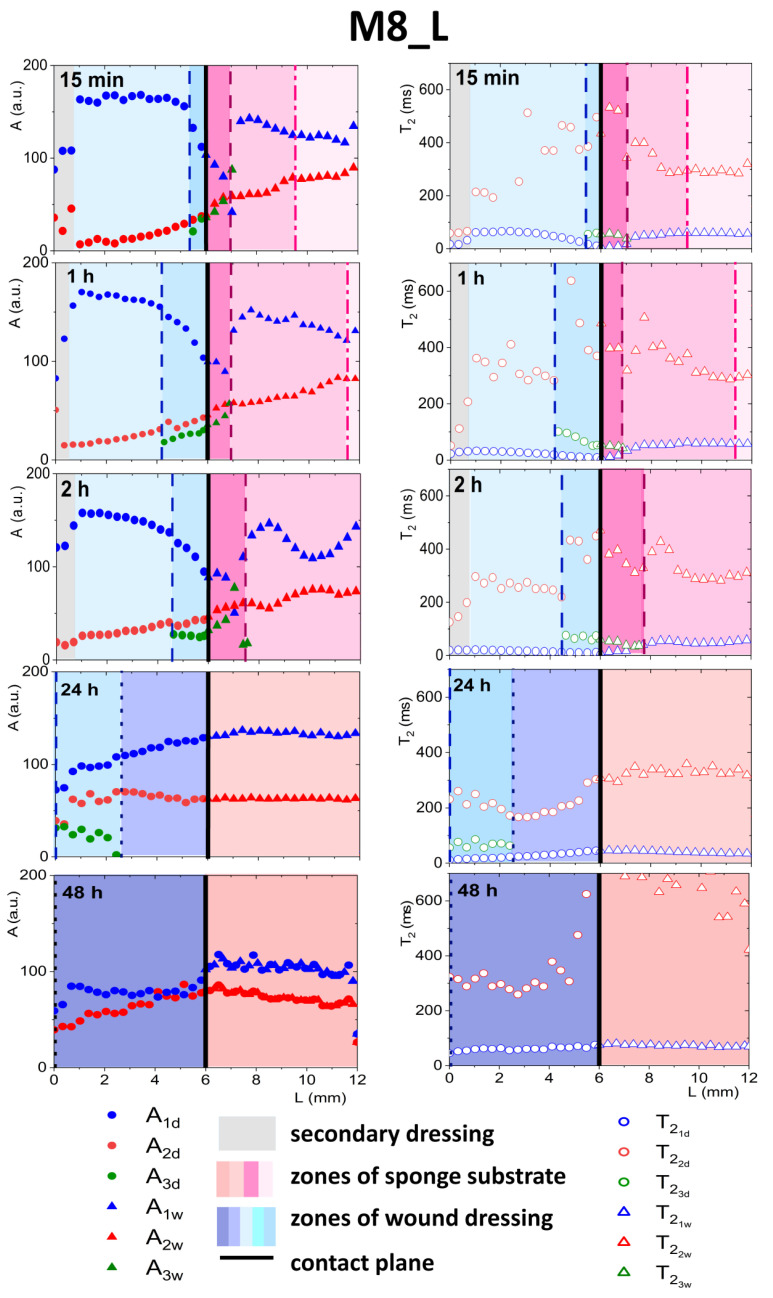
Parameter profiles obtained by MRI, relaxation time (*T*_2_) or signal amplitude (*A*), for M8_L for the whole in vitro wound dressing stack model, when the sponge medium solution was PG.

As was explained previously, initially, two components inside the wound dressing were detected before application—the first originating from water and the second originating from PG (see Figure 3). In both dressings, after contact with PG, a third distinct exponential component appeared in the MR signal at spatial positions around the contact plane (see Figure 6 and Figure 7). In M8 dressing, this region expanded gradually and at 24 h three components in the MR signal were present across the whole wound dressing model stack including the sponge substrate (Figure 6). In M8_L, this zone moved across the dressing towards the top of the dressing and at 24 h only two components in the MR signal were present (Figure 7). In both cases, it was associated with the diffusion of PG into the dressing as the initial concentration of PG at sponge substrate was much higher than at the dressing side.

#### 3.4.1. Detailed Description of M8 Formulation Results

As a result of the penetration of PG into the dressing (Figure 6), the relaxation time of the water component $T_{2_{1d}}$ was shortened and the relaxation time $T_{2_{2d}}$ of the PG component was extended. The new component had a *T*_2_ relaxation time of about 50 ms. This third component could not be regarded as an effect of improper signal fitting—three distinct components were clearly visible in the continuous *T*_2_ distribution obtained after Laplace inversion of the signal taken from the ROI localized around the contact plane, i.e., at *L* ca. 6 mm (Figure 5a).

The spatial distribution of amplitudes also changed as a consequence of diffusional processes. PG entered the dressing while water diffused to the sponge substrate. This was reflected in the amplitude of the components—the *A*_2*d*_ amplitude increased as the *A*_1*d*_ decreased toward the contact plane (see Figure 6). The $T_{2_{1d}}$ relaxation time was shortened because part of the free water left the dressing and diffused into the sponge substrate.

Only water molecules strongly interacting with the PVA matrix remained in the wound dressing (their relaxation time was shorter and the *A*_1*d*_ amplitude decreased). The $T_{2_{2d}}$ value of the PG component increased as PG particles originally existing in the dressing agglomerated with PG particles diffusing into the dressing. A possible explanation for this was that the PG molecules reached the “gaps” and “pores” in the PVA network and filled new, ever-smaller free spaces. Probably, PG protons were exchanged with PG protons that left the dressing and were hydrated. This increased their relaxation time. According to Zhou et al., protons derived from bulk water have a higher mobility and their mobility decreases with an increase in the molar concentration of PG in the mixture, especially in the range of low PG concentrations [30].

The appearance of the third signal component at 15 min was connected with a kind of front in the dressing at *L* ca. 4.0 mm (marked with vertical dashed line in Figure 6). This was associated with a change in the spatial trend of a shorter water component (*A*_1*d*_) and a decrease, towards the contact plane, in $T_{2_{1d}}$. This front was moving with time towards the dressing top—*L* = 4 mm at 15 min, *L* = 3.5 mm at 1 h, *L* = 3 mm at 2 h. At 24 h three components existed across the dressing. The diffusional processes in the M8 dressing took quite a long time, much longer than when the medium in the sponge substrate was serum (FBS). Even comparing results obtained at 24 h and 48 h, changes were observed (*T*_2_ of the longest component decreased). Finally, the system stabilized in terms of *T*_2_ at relatively constant levels with the longest *T*_2_ component of 250 ms, the intermediate of 50 ms and the shortest of 13 ms.

Starting from the contact plane and moving to the right, i.e., deeper into the sponge substrate, a shortening of the component related to small pores $T_{2_{1w}}$ in the sponge (compared to the value at the end of the sponge, e.g., around *L* from 10 to 12 mm for measurements up to 2 h). The appearance of an additional component with an intermediate relaxation time $T_{2_{3w}}$ ≈ 45 ms with a simultaneous increase in amplitude towards the right of the contact plane was observed. It could be concluded that the short PG component present in the sponge before application of the dressing split into two parts. The component related to PG in large pores was also changed, and the closer to the contact, the higher the $T_{2_{2w}}$. The amplitude of the long component from PG from the sponge substrate *A_2w_* was comparable to the new additional component *A*_3*w*_. The observed trends result from the penetration of substances from the dressing into the sponge substrate. The increase in *T*_2_ of the long component and the splitting of the short component were related to the mixing of PG existing already in the sponge substrate with water, PG and PVA that diffused from the dressing. The position the substances reached shifted to the right, deeper into the substrate. This position (front) is marked with a dashed line in Figure 6. Successively, for 15 min after application, the position of this front was *L* = 7.4 mm, for 1 h *L* = 8.4 mm, for 2 h *L* = 9.3 mm. Another zone in the substrate was distinguished deeper in the substrate. It was not possible to distinguish the third component in this zone, but changes in relaxation times and amplitudes related to the diffusion of substances exiting the dressing were already visible (the end of this zone was marked with a dash-dotted line). After 24 h, three components in the entire volume of the sponge substrate were observed but the spatial profiles were flattened. After 48 h, there were still changes in amplitudes. At the bottom of the sponge (*L* > 10 mm), there were still three proton fractions with different relaxation times, although their amplitudes were of similar values. Zhou et al. have emphasized a relatively slow chemical exchange between hydroxyl groups. This could explain the difference between profiles obtained at 24 h and 48 h after dressing application. In mixtures of PG with water, Zhou et al. have detected several proton fractions of different molecular mobilities depending on the molar concentration of PG [30]. They have observed the separate peaks in the magnetic resonance spectrum belonging to water and PG hydroxyls (triplet and doublet splitting).

#### 3.4.2. Detailed Description of M8_L Formulation Results

In the case of M8_L, as mentioned above, the third exponential component also appeared (Figure 7), but the penetration of PG into the dressing was slower than in M8. This was due to a more compact structure and smaller pores in the material [16]. As for the M8 formulation, one could identify the front in the wound dressing, marking the appearance of the third component (*L* ca. 5.5 mm at 15 min marked with dashed line). Inside a zone in the dressing characterized by the coexistence of three components, several features could be observed, i.e., a decrease in amplitude of water components (*A*_1*d*_), and an increase in the amplitude of PG components (*A*_2*d*_), together with a decrease in $T_{2_{1d}}$ relaxation time and an increase in $T_{2_{2d}}$. At the front, marking beginning of the zone, the amplitude of the water component also changed its spatial characteristics (i.e., it became steeper). The spatiotemporal evolution of this zone was different to that in the M8 case. At 24 h, a spatial shift of the three components zone towards the top of the dressing (*L* in the range of 0 ÷ 2 mm) was observed. For *L* > 2 mm, the properties of the dressing in terms of signal components returned to its original two exponential characteristics. Consequently, at 48 h, two components in the MR signal across the dressing were present. Taking into account the spatial changes of the *A*_1*d*_ amplitude after 24 h of the experiment, i.e., higher at the contact plane than at the initial *L* values, a continuous water diffusion outside the dressing and its replacement with hydrated PG could be assumed [30]. At 48 h, after flattening profiles across both dressing and the substrate, an increase in *T*_2_ relaxation time of both components was observed. The value of *T*_2_ of the long component of the dressing $T_{2_{2d}}$ was approximately 300 ms and the shorter relaxation time,$\;T_{2_{2d}}$, was approximately 60 ms. The amplitude ratio was also changed. Probably, only the components of free PG (characterized by longer *T*_2_) and the component originated from both bound PG and water entrapped in the network (shorter *T*_2_) could be detected.

At the dressing/sponge substrate interface, the same medium exchange takes place as for the M8, but the results suggest that more water was moving out of the dressing. After 24 h, the third component in the substrate was not observed. After 48 h, the *T*_2_ of the longer component increased significantly, possibly due to PG hydration. PG is found to be readily hydrated, and the most common hydration interactions occur through both the hydroxyl groups of PG and the alkyl groups, which are typically considered hydrophobic [31].

Moving fronts or evolving layers has been previously detected using MR imaging in pharmaceutical polymeric matrices intended for use as controlled or modified release dosage forms [32,33,34,35,36]. It has also been shown that the development of a particular internal structure in terms of layers and subsequent structure evolution influence the functional properties of the system [37,38,39]. These works suggest that the application of the approach to wound dressing materials could improve the rational design of such materials. The developed wound dressing stack in the current version could be used for investigation of wound dressings in several applications, e.g.,: (1) for assessment of newly-designed materials in terms of their usefulness as wound dressings; (2) for assessment of the potential behavior of the hydrogel dressing when applied to a wound; (3) for matching the newly designed material with the type of the wound (amount of exudate) that it is to be applied to; (4) for assessment of the dressing’s ability to both absorb exudate and release moisture to the application surface.

## 4. Conclusions

In this study, a simplified in vitro wound dressing stack model was developed to mimic two different interfacial environments: (1) wound dressing contact with wound exudates in vivo; and (2) wound dressing contact with acceptor fluid during pharmaceutical in vitro drug release test in diffusion cell apparatus. Two PVA-based dressings were examined in situ using two substrate environments (FBS and PG) by magnetic resonance imaging inside the developed stack model. Multi-exponential analysis of the relaxometric data allowed to distinguish signal components originating from water and PG in various environments. The components were related to bidirectional mass transport (water, PG) in the dressing/substrate system and subsequent changes of physicochemical properties of the dressing and its adjacent substrate. They enabled the characterization of the spatial structure of the wound dressing and its spatiotemporal evolution in terms of moving fronts and corresponding layers. A fast and relatively simple evolution of the dressing properties was observed when using FBS as substrate medium—PG leakage-related changes were observed inside the dressing. When using PG as a substrate medium, slower evolution of the dressing properties was observed with a much more complicated dressing structure in terms of fronts and layers. Two types of process were observed after hydrogel wound dressing application: (1) spatial changes that could be described using moving fronts and layers resulting in flattening of the spatial *T*_2_ and *A* profiles across the dressing (or substrate); (2) subsequent changes occurring in the whole volume of the dressing (or substrate), excluding a narrow contact zone.

## Figures and Tables

**Table 1:** Average values of the *A* and *T*_2_ parameters as measured across the hydrogel wound dressing.

	*A*_1*d*_ (a.u.)	$T_{2_{1d}}\;(\mathrm{ms})$	*A*_2*d*_ (a.u.)	$T_{2_{2d}}\;(\mathrm{ms})$
M8	195± 12	48.9 ± 2.6	8.0± 1.1	394 ± 157
M8_L	199 ± 3.6	29.7 ± 0.4	14.2 ± 1.1	296 ± 59

## Data Availability

The processed data required to reproduce these findings is available from the authors upon reasonable request.
